# Renal protective potential of pentoxifylline, chlorpromazine, and lovastatin in ischemia-reperfusion injury: An experimental study

**DOI:** 10.1371/journal.pone.0308649

**Published:** 2024-10-16

**Authors:** Daniel Peixoto Pereira, Brunna Silva Moreira, Marcela Aldrovani Rodrigues, Larissa Fernandes Magalhães, Luana de Oliveira Branco, Natani Silva Reis, Sofia Borin-Crivellenti, Leandro Zuccolotto Crivellenti

**Affiliations:** 1 Veterinary Teaching Hospital/ Animal Science Graduate Program, Franca University (UNIFRAN), Franca, São Paulo, Brazil; 2 Graduate Program in Veterinary Science, Universidade Federal de Uberlândia (UFU), Uberlândia, Minas Gerais, Brazil; Dr. Anjali Chatterji Regional Research Institute for Homeopathy, INDIA

## Abstract

This study aimed to evaluate the ability of pentoxifylline when compared to lovastatin and chlorpromazine as nephroprotective substances in cases of renal ischemia and reperfusion syndrome (IRI). A total of 36 adult male animals were randomly allocated into four groups (untreated control group, pentoxifylline group, lovastatin group, and chlorpromazine group), each consisting of nine animals. All groups were submitted to experimental ischemia and reperfusion procedures. The animals were evaluated 24, 72 and 120 hours after IRI, including physical examinations, serum urea and creatinine measurements, as well as histopathological, morphometric, and stereological analyses of the renal tissue. Results indicated that 24 hours after IRI, only chlorpromazine was effective in controlling azotemia. At the 72-hour mark, both chlorpromazine and pentoxifylline exhibited efficacy. After 120 hours, all three substances demonstrated renal protective qualities. Pentoxifylline was the most effective in preserving the structural integrity of kidney tissue, followed by chlorpromazine. In conclusion, all three treatments (pentoxifylline, chlorpromazine, and lovastatin) were effective. Pentoxifylline proved to be promising in the response against acute tubular necrosis, although chlorpromazine presented earlier renoprotective effects in terms of maintaining renal function.

## Introduction

Ischemia and reperfusion syndrome (IRI) is one of the main causes of acute kidney injury, especially after hypovolemic shock, cardiovascular surgery and kidney transplantation [1, 2]. The mechanism proposed to explain ischemic injury includes alterations in nucleotide metabolism, with a reduction in ATP production, which leads to increased expression of epithelial adherence molecules and production of cytokines and tumor necrosis factor (TNF), and leukocyte attack on the renal parenchyma [3].

IRI results in capillary endothelial dysfunction and tubular necrosis, leading to acute kidney injury due to ischemia, inflammatory reaction caused by cytokine activation, recruitment of the macrocytic-phagocytic system, synthesis of free radicals and neutrophil activation [4]. the latter being the main cause of transplanted organ failure [1].

Currently, IRI models are evaluated morphologically using semi-quantitative histological methods. Limitations of this approach include lack of reproducibility, interobserver variability and limited sample analysis [5].

Drugs have been explored to reduce tissue damage associated with IRI. Chlorpromazine, for instance, still requires studies into its mechanism of action despite its nephroprotective action [6]. It should be highlighted that the nephroprotective effect of chlorpromazine only occurs when it is administered before the blood supply to the tissue stops [7]. Another drug used is lovastatin, which has a positive effect on renal circulation by increasing blood flow to the organ, even in nephropathic animal [8].

Pentoxifylline, a methylxanthine derivative, is a drug that has not been widely explored in the clinical management of IRI. It has de potential to increase blood flow through peripheral vessels and tortuous capillaries, as it alters the ability of erythrocytes and granulocytes to deform, decreases fibrinogen levels and thromboxane release, inhibits platelet aggregation, and increases prostacyclin levels [9, 10]. An *in vitro* study has found that pentoxifylline has an inhibitory effect on the production of TNF-α by monocytes and T lymphocytes [10].

The hypothesis of this study is that pentoxifylline has nephroprotective potential against ischemic acute kidney injury since it seems to have peripheral hemodynamic effects and favors the maintenance of a cytoplasmic environment suitable for maintaining intracellular integrity. This study investigated whether pentoxifylline is nephroprotective and its effects compared to the drugs chlorpromazine and lovastatin.

## Material and methods

### Animal subjects and experimental approach

A total of thirty-six adult male Wistar rats, with an average weight of 229g ± 18g, were used for this study. Ethical clearance for the study procedures was approved by the Ethics Committee for Animal Use, CEUA-UNITRI, protocol number 849855/2013.

Prior to the procedures, the rats were kept for 15 days in an air-conditioned bioterium and handled by the research team in order to adapt and reduce stress from the environmental change. During the experiment, the rats were given water and industrialized food ad libitum.

Each rat underwent a physical examination, and its body condition was assessed according to a score of 1–5 (1—cachectic, 3—ideal, 5—obese), degree of dehydration through CPT and skin elasticity, mucous membrane coloration and food intake through daily weighing of feed and water volume. The animals were randomly assigned to four groups of equal number (n = 9), called the Control group (CT), the Pentoxifylline group (PTX), the Chlorpromazine group (CLOR) and the Lovastatin group (LOV).

The procedures for inducing IRI were identical for all groups. The PTX group received oral administration of pentoxifylline at a dose of 40mg/kg/day for 10 days before induction of ischemia and renal reperfusion. The CLOR group received intravenous injection of 3mg/kg chlorpromazine through the external jugular vein, 15 minutes before ischemia. The LOV group received oral doses of lovastatin of 15mg/kg/day for a 10-day period before the procedure. The CT group received saline solution, and manipulation to maintain similarity between stressors.

Each group was randomly divided into three sub-groups, each consisting of three animals. These sub-groups were individually housed in separate cages, marked as 24h, 72h, and 120h to identify the evaluation points after the ischemia-reperfusion intervention.

### Surgical procedure

The animals were induced and maintained under anesthesia using isoflurane (Isoforine—Cristália—Itapira—Brazil), vaporized in pure oxygen through a mask in a semi-open system.

Each individual was prepared by trichotomy in the abdominal region, followed by antisepsis using 70% alcohol (Rialcool 70—Rioquímica—São José do Rio Preto—Brazil) and povidone-iodine solution (Riodeine—Rioquímica—São José do Rio Preto—Brazil). Additionally, fentanyl citrate (Fentanest—Cristália—Itapira—Brazil) was administered intramuscularly at a dose of 0.025 mg/kg.

A pre-umbilical median celiotomy was performed, and blood samples were collected by venipuncture of the caudal cava vein for subsequent laboratory analysis. To ensure that the observed cytoprotective effects of the administered drugs were not influenced by controlled hypothermia, normothermic ischemia was used. Ischemia was induced by clamping the left renal artery with 4 cm Dieffenbach bulldog clamps. Following this, myorrhaphy and dermorrhaphy were performed, and the animals were placed in individual boxes for 60 minutes. They were then re-anesthetized with isoflurane, the abdominal cavity was reopened, and blood flow was re-established by removing the vascular clamp, followed by a nephrectomy of the right kidney. The muscle and skin were closed by a single continuous stitch using 4–0 monofilament nylon thread (Nylon—Shalon fios cirúrgicos—São Luís de Montes Belos—Brazil).

After recovery from anesthesia, the animals were kept to their respective cages. Analgesia was given with tramadol hydrochloride (Tramadon—Cristália—Itapira—Brazil) at a dose of 5mg/kg, intramuscularly, every 12 hours for up to 3 days, depending on time of euthanasia.

### Sample collection

At the 24-hour of IRI, three animals from each group were anesthetized with isoflurane inhalation via a mask. Following surgical field preparation, a celiotomy was performed to collect blood by puncture of the caudal vein, alongside with a left nephrectomy. Then, while the animal still anesthetized, euthanasia was performed with intracardiac administration of a 19.1% potassium chloride solution (potassium chloride—Isofarma—Eusébio—Brazil).

Blood samples were stored in tubes equipped with serum separator gel, while the collected kidney was preserved in a 10% formaldehyde solution (37% formaldehyde solution—DGL Indústria e Comércio—Franco da Rocha—Brazil). The same procedures were performed on the other animals after 72-hour and 120-hour intervals, ensuring consistency throughout the data collection process.

### Laboratorial analysis

#### Biochemical blood analysis

The enzymatic calorimetric method was used to assess serum urea and creatinine using the Urit-8021A equipment.

#### Histopathological, morphometric, and stereological analysis of kidneys

The collected kidney was kept in 10% formalin for 24 hours, embedded in paraffin and sectioned to a thickness of 4 μm. These sections were then stained with Hematoxylin-Eosin (HE). Each sample was given a numerical code, with no group or subgroup identification.

Histopathological analyses were carried out blindly and subjectively by a single pathologist. This evaluator assessed the type of tubular lesions and rated them based on its intensity: absent (0), mild (1), moderate (2), and severe (3), based on methodologies used in previous studies [11].

A total of twenty-five images were captured from each kidney section (3 per animal) using an optical microscope (Zeiss, Oberkochen, Germany) with a high-resolution digital color video camera (Zeiss) and transferred to a computer platform, where they were digitized for software-assisted morphometric and stereological analyses. The microscope’s operational settings remained uniform for all the samples (20x Plan Neofluar objective, illumination by halogen lamp, focal planes calibrated through Köhler illumination).

For morphometric analyses, Image J® software (NIH, Bethesda, USA) was used to quantify the area of tubular necrosis (μm^2^). Stereological evaluations were conducted according to Cavalieri’s principle. Estimation of the cortical-medullary ratio was performed, as per established recommendations [12].

The cortical-medullary ratio and glomerular volumetric density (Vv[Glom]) were obtained using the point-counting technique, which was implemented with the assistance of the M42 test system. This system was overlaid onto kidney images using the STEPanizer stereological tool, version 1.0 (Bethesda, USA).

#### Statistical analysis

For statistical analyses, the JMP software (SAS Institute Inc., Cary, NC, USA) and GraphPad Prism 5 was used. The normality of data distribution was tested by the Kolmogorov-Smirnov test. In cases where data exhibited normal distribution, a one-way analysis of variance (ANOVA) was used, followed by Tukey’s post hoc analysis. Since histopathological analysis score has not fit normal distribution, statistical evaluations were performed using the Kruskal-Wallis nonparametric test, followed by Dunns’ post hoc test. Statistical significance was considered as P < 0.05.

## Results

Sixty minutes after ischemia, a distinctive cyanotic and congested appearance was observed in the kidneys of all groups. In the first 24 hours after reperfusion, animals in the CT group presented a gradual decline in physical activity (n = 9, 100%) and appetite (n = 6, 66.7%). In contrast, normal behavior was observed in all animals from the other groups. After 72 hours, animals from the CT (n = 6, 100%), LOV (n = 6, 100%), CLOR (n = 6, 100%), and PTX 72h (n = 6, 100%) groups presented prostration and hyporexia, with the latter two groups being more intense. After 120 hours, the animals treated with all three drugs exhibited clinical stability, with normal appetite, active behavior, and general appearance. By contrary, all animals in the control group presented significant weight loss, intense prostration, dehydration, and anorexia.

**Fig 1:**
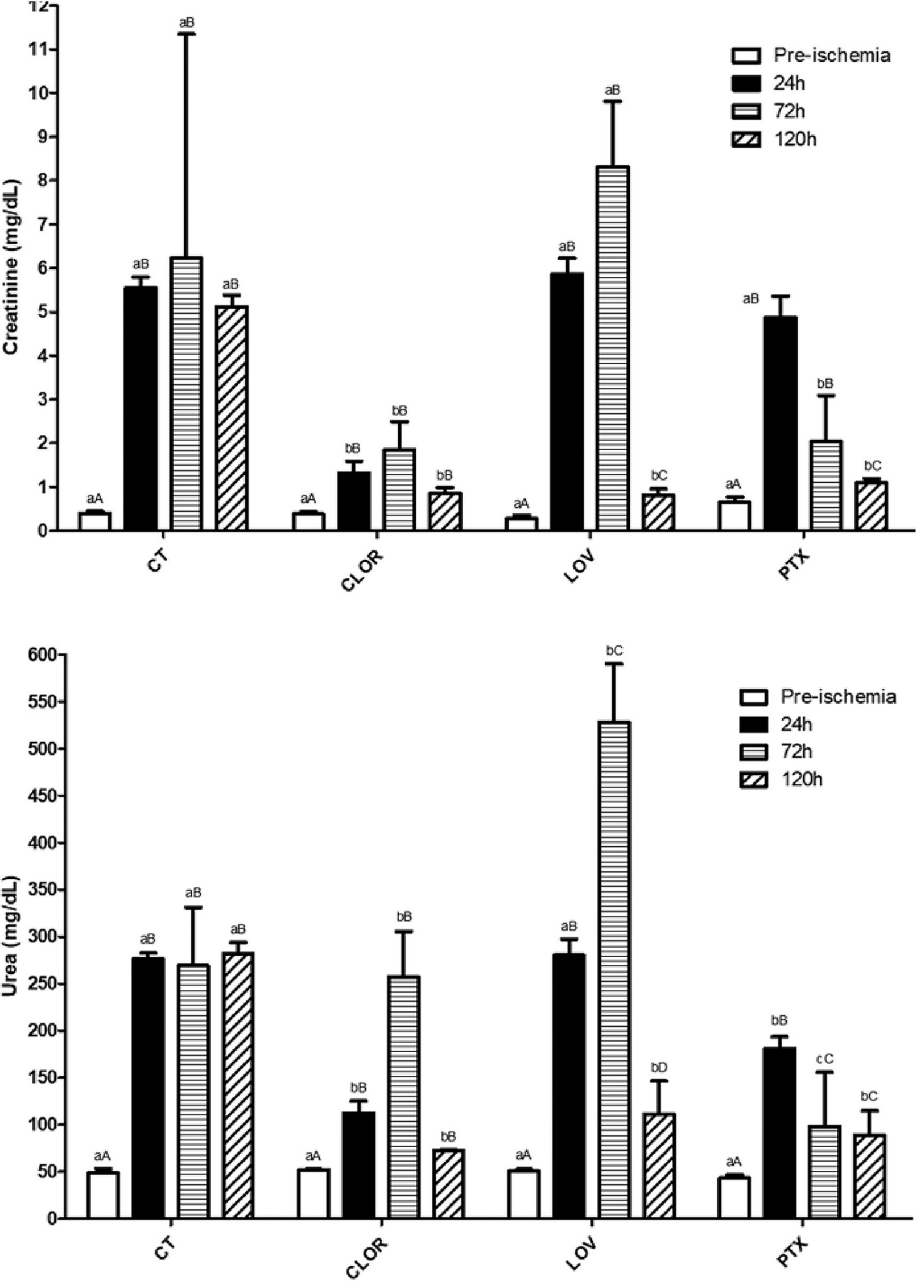
Graphic 1. Results of creatinine and urea dosages for each group and per evaluation period. Different lowercase letters in the same row indicates a statistically significant difference between the groups and different uppercase letters in the same column indicates a statistically significant difference between time periods (P < 0.05).

Azotemia was observed in the CT, LOV, and PTX groups. However, the increase in creatinine and urea levels was significantly lower in the CLOR group at 24 hours (Fig 1) (P<0.05). After 72 hours, the CLOR group presented mild azotemia, meanwhile levels were lower in the PTX group. At the same time, both the CT and LOV groups had increased creatinine and urea levels when compared to the initial 24 hours (P<0.05). At 120 hours, a significant reduction in creatinine and urea concentrations was observed in the treated groups compared to the CT group (Fig 1) (P<0.05).

**Fig 2:**
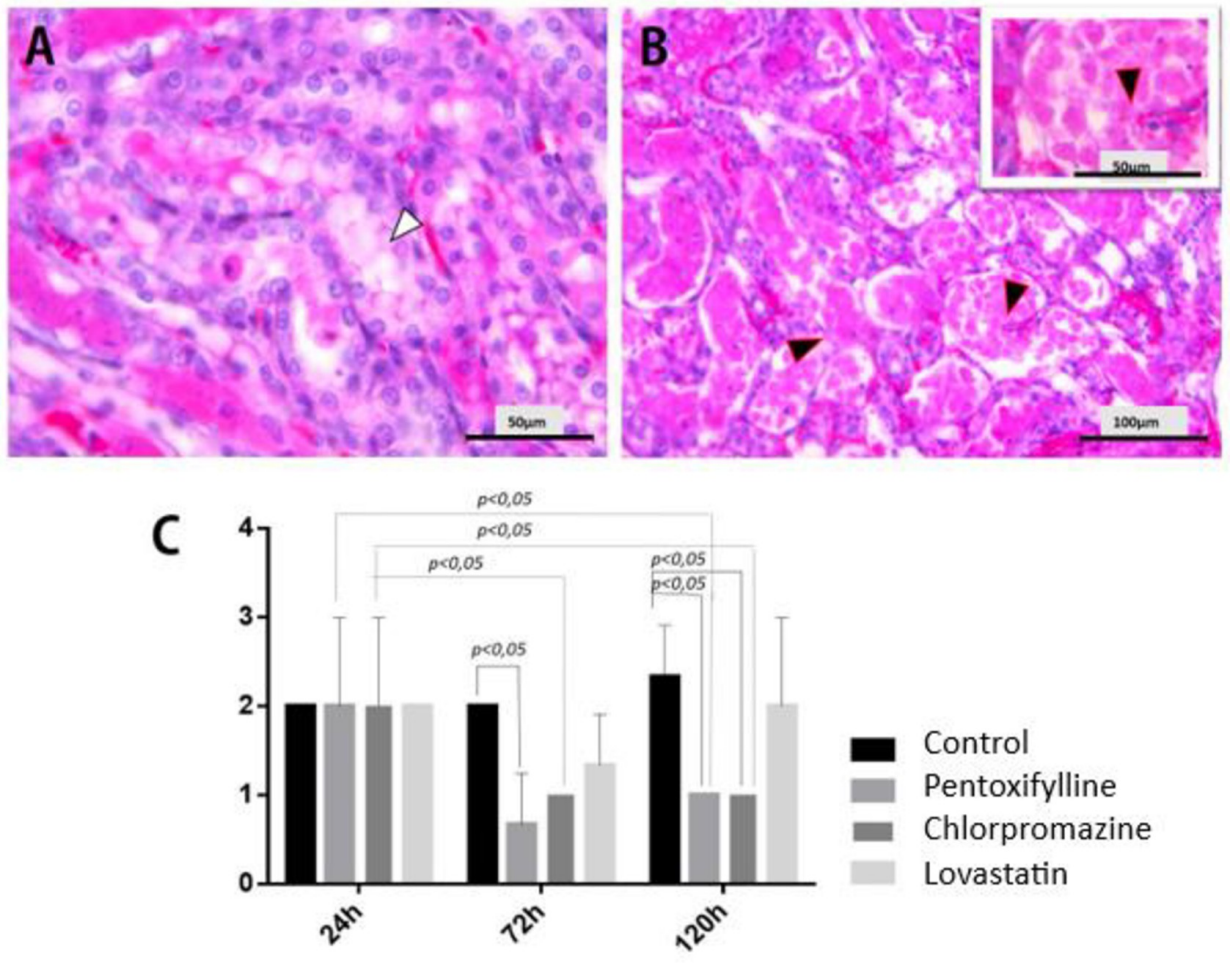
Photomicrographs illustrating tubular alterations from IRI. (A) Tubular degeneration (white arrow), indicating non-lipid cytoplasmic vacuolization within tubular cells. (B) Tubular necrosis (black arrow) after IRI, presenting discrete nuclear traces in the right corner, while most cells lack nuclei. (C) Bar graph indicating tubular necrosis scores.

Histopathological analysis demonstrated renal tubular degeneration across all study groups and evaluation periods (Fig 2A), with no statistically significant differences between them (P>0.05). Following 24 hours of ischemia and reperfusion, it was observed a presence of moderate tubular necrosis in all groups (Fig 2B). After 72 hours, the severity of necrosis reduced from moderate to mild (P<0.05) in the PTX group, maintaining this status until the 120-hour time evaluation. The intensity of necrosis in the CLOR group at 120 hours demonstrated a considerable reduction compared to the CT group (P<0.05). In contrast, the LOV group did not differ from the CT group (P>0.05).

The morphometric study revealed that, following the ischemia and reperfusion procedures, the CT group had more extensive areas of tubular necrosis (42.79 ± 1.88% of the total observed area per microscopic field studied). In contrast, the PTX (35.28 ± 3.13%), CLOR (37.80 ± 2.61%), and LOV (38.37 ± 2.42%) groups had relatively smaller necrotic areas (P<0.05). Further examinations reveal that, during the 120-hour evaluation, the renal tubular area containing necrotic cells increased to 51.26 ± 3.76% in the CT group and reached 43.14 ± 1.76% in the LOV group (P<0.05). By contrary, neither the PTX nor the CLOR groups had a comparable increase (P>0.05).

**Fig 3:**
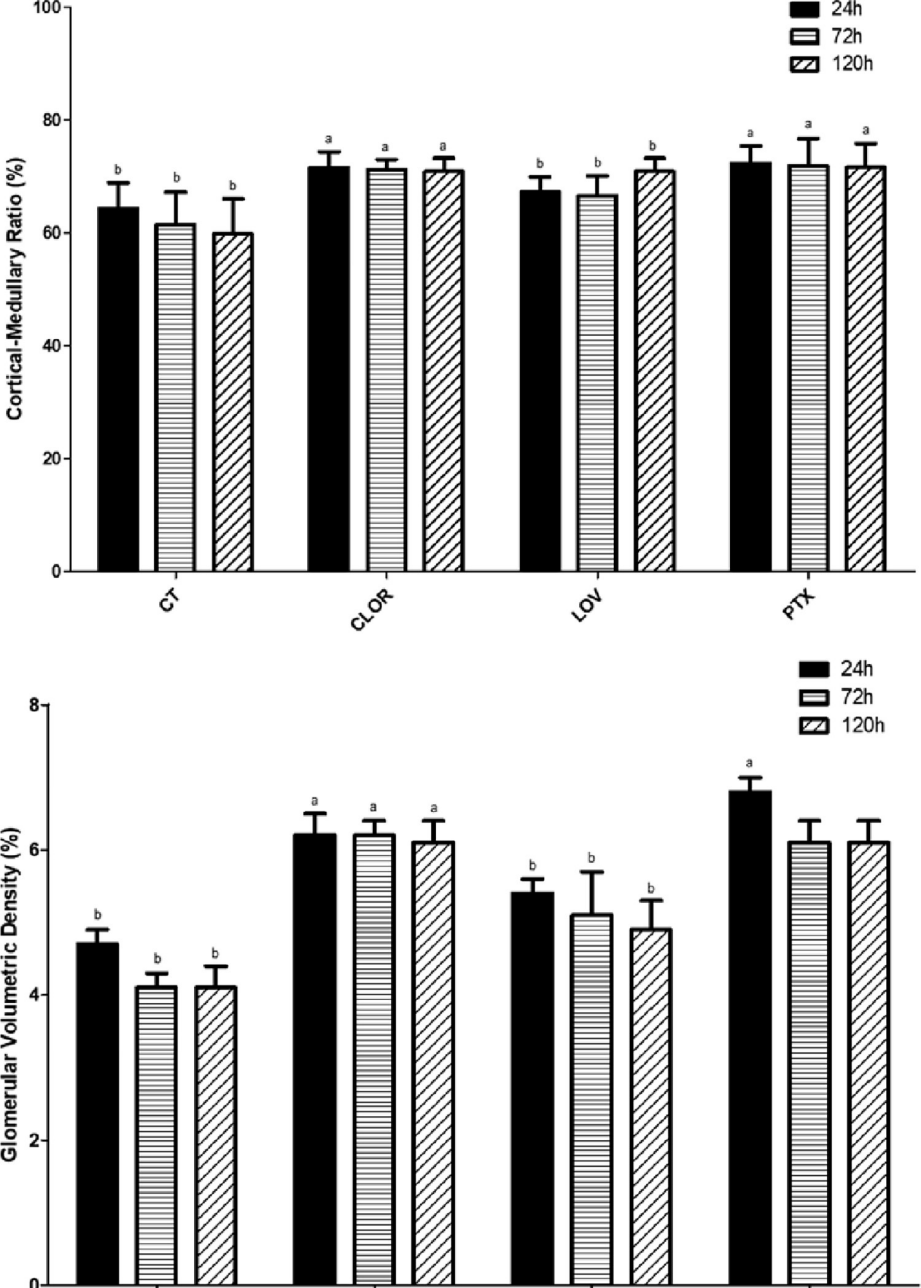
Graphic 2. Stereological data from rat kidneys in the study groups. CT, untreated control; PTX, treated with pentoxifylline; CLOR, treated with chlorpromazine; LOV, treated with lovastatin. Vv[Glom], glomerular volumetric density; Different lowercase letters in the same column indicates a statistically significant difference between the groups.

The stereological analyses reveal significant differences (P<0.05) in both the cortical-medullary ratio and the glomerular volume density between the PTX and CLOR groups when compared to the CT and LOV groups (Fig 3).

## Discussion

In this study, a comparative evaluation of two drugs already integrated into clinical practice (CLOR [7, 13] and LOV [14, 15]) was performed, alongside an experimental drug, PTX [16–18], to analyze their potential nephroprotective capacities against IRI. The earlier nephroprotection observed in PTX and CLOR groups is probably related to the vasoactive effects stemming from phosphodiesterase blockade [9, 16, 17] and α1 adrenergic blockade [7], respectively. These mechanisms establish a quick blood supply during hypoxemic processes.

The proposed treatments (PTX, CLOR, and LOV) proved to be effective. The best nephroprotective response observed in the initial 24 hours in the CLOR group, attributed to its membrane-stabilizing capability, preventing calcium-dependent phospholipid alterations, calcium channel blocking, and reduction in mitochondrial membrane degradation, reactivating cellular energy production a few moments after reperfusion [13, 19]. The sustained effect of CLOR is probably related to its ability to reduce intracellular toxic aldehydes, minimizing the arachidonic acid cycle and inflammatory chemotactic factors. Moreover, by inhibiting Na+/K+ ATPase pump activity and blocking calcium channels, CLOR reduces hydrogen release into the respiratory chain, maintaining intracellular pH10. These factors together contributed to restoring urea and creatinine concentrations to baseline levels 120 hours post-procedure, presenting better results than the previously proposed indicating recovery after 7 days of IRI [13]. Thus, the drug proves beneficial in both reduction of initial degradation and secondary inflammation process.

While Pentoxifylline exhibited an improvement in azotemia after 72 hours, similar to previous findings [16], its nephroprotective action had not prevented the initial ischemic lesions from occurring. This suggests that in the absence of renal flow, PTX’s efficacy is limited due to its restricted penetration into the ischemic cell bed [16, 17]. The PTX’s efficacy post 72 hours may be attributed to its intracellular mechanism of action. Unlike CLOR, PTX may not target primary activation systems in the membrane; instead, its action occurs slightly later in the intracellular environment, generating cAMP accumulation, which reduces TNFα formation [20] and mast cell degranulation [21], thereby mitigating pro-inflammatory effects.

In the LOV group, the reduction in azotemia occurred later among the drugs tested, probably due to its impact on reducing proteinuria [14, 22] and reduction in tubular damage. This effect is consistent with findings in early-stage diabetic nephropathy, as reported in a meta-analysis of 2866 individuals [14]. Evidently, while lovastatin does not inhibit acute kidney injury resultant from ischemia, it may help recovery during the initial damaging process [8], mainly due its oxidative stress control [19].

In terms of kidney damage, none of the drugs prevented tubular degeneration even 120 hours post-IRI. However, both chlorpromazine and pentoxifylline successfully reduced tubular necrosis. Furthermore, the PTX and CLOR groups presented higher cortical-medullary ratios, Vv [Glom], and VWGV compared to the CT and LOV groups, indicating a correlation between chlorpromazine and pentoxifylline administration and reduced renal lesions.

These results indicate that pentoxifylline demonstrates an early nephroprotective effect, complementing the favorable effects of chlorpromazine. This may be associated to pentoxifylline’s capacity to inhibit the adhesion and activation of dendritic cells, lymphocytes, and natural killer cells [23]. Additionally, pentoxifylline’s inhibition of TGF-β and connective tissue growth factor (CTGF) synthesis probably contributes to reduced collagen synthesis, renal fibrosis [24, 25], and tissue protection against IRI, generating a higher recovery potential post-ischemic tubular necrosis.

It is worth noting that the comparatively minor protective effect of LOV on tubular necrosis in the initial 120 hours after IRI contrasts with studies highlighting its beneficial results, including the reduction of endothelial dysfunction [15] and the inhibition of cholesterol deposition in the proximal tubules after acute tubular injury due to ischemia [26]. This discrepancy may probably be related to the comparison with the control group, which presented higher results, or the extended medication use, with longer periods in other studies (5mg/kg per day for 16 weeks), which may be less viable in routine kidney transplantation, requiring more agility drug intervention.

## Conclusion

PTX demonstrated promise by achieving a favorable response against acute tubular necrosis when considering their distinct mechanisms of action. It is proposed that the combination of PTX and CLOR may present greater benefits in situations involving programmed IRI.

## Supporting information

S1 Data Statistical data for supporting information. (DOCX)

S2 Data Descriptive statistical data. (XLSX)
