# Supplementary material for: Renal protective potential of pentoxifylline, chlorpromazine, and lovastatin in ischemia-reperfusion injury: An experimental study
Source: PLoS One. 2024 Oct 16;19(10):e0308649. doi: 10.1371/journal.pone.0308649 (PMC11482703; doi:10.1371/journal.pone.0308649)
Supplement: S1 Data — (DOCX) [file pone.0308649.s001.docx]

**Supporting information for Graphic 1.** Means and standard deviation of creatinine and urea dosages for each group and per evaluation period.

|  |  | CT | CLOR | LOV | PTX |
| --- | --- | --- | --- | --- | --- |
| Pre-ischemia (n=9) | Creatinine | 0.40 ± 0.05^aA^ | 0.38 ± 0.05^aA^ | 0.27 ± 0.08^aA^ | 0.65 ± 0.12^aA^ |
|  | Urea | 48.65 ± 4.44^aA^ | 52.24 ± 0.27^aA^ | 50.76 ± 1.63^aA^ | 43.31 ± 3.4^aA^ |
| 24h (n=3) | Creatinine | 5.55 ± 0.25^aB^ | 1.32 ± 0.27^bB^ | 5.86 ± 0.37^aB^ | 4.87 ± 0.49^aB^ |
|  | Urea | 276.33 ± 6.03^aB^ | 112.56 ± 12.42^bB^ | 280.26 ± 17.06^aB^ | 180.80 ± 12.51^bB^ |
| 72h (n=3) | Creatinine | 6.23 ± 5.12^aB^ | 1.85 ± 0.64^bB^ | 8.32 ± 1.49^aB^ | 2.04 ± 1.05^bB^ |
|  | Urea | 269.30 ± 61.85^aB^ | 257.26 ± 48.09^aC^ | 527.86 ± 62.30^bC^ | 98.13 ± 57.75^cC^ |
| 120h (n=3) | Creatinine | 5.12 ± 0.26^aB^ | 0.86 ± 0.12^bB^ | 0.81 ± 0.15^bC^ | 1.09 ± 0.10^bC^ |
|  | Urea | 282 ± 11.79^aB^ | 72.56 ± 0.99^bAB^ | 111.36 ± 35.03^bD^ | 88.93 ± 25.85^bC^ |

Different lowercase letters in the same row indicates a statistically significant difference between the groups and different uppercase letters in the same column indicates a statistically significant difference between time periods (P < 0.05)

| **Supporting information for Graphic 2.** Means and standard deviation of stereological data for rat kidneys that composed the study groups | | | | | | | | | | | | |
| --- | --- | --- | --- | --- | --- | --- | --- | --- | --- | --- | --- | --- |
| **Stereological parameter** | **CT** | | | **PTX ^*, †^** | | | **CLOR ^*, †^** | | | **LOV** | | |
|  | **24h** | **72h** | **120h** | **24h** | **72h** | **120h** | **24h** | **72h** | **120h** | **24h** | **72h** | **120h** |
| **Cortical-medular ratio (%)** | 64.3 ± 4.6 | 61.5 ± 5.7 | 59.8 ± 6.2 | 72.3 ± 3.1 | 71.9 ± 4.8 | 71.6 ± 4.2 | 71.5 ± 2.9 | 71.2 ± 1.8 | 70.9 ± 2.3 | 67.2 ± 2.7 | 66.6 ± 3.5 | 66.8 ± 3.9 |
| **Vv[Glom] (%)** | 4.7 ± 0.2 | 4.1 ± 0.24 | 4.1 ± 0.3 | 6.8 ± 0.2 | 6.1 ± 0.3 | 6.1 ± 0.3 | 6.2 ± 0.3 | 6.2 ± 0.2 | 6.1 ± 0.3 | 5.4 ± 0.2 | 5.1 ± 0.6 | 4.9 ± 0.4 |
| CT, untreated control; PTX, treated with pentoxifylline; CLOR, treated with chlorpromazine; LOV, treated with lovastatin.  Vv[Glom], glomerular volumetric density; VWGV, weighted mean glomerular volume.  *, p < 0,05 versus CT group (all evaluation times)  †, p < 0,05 versus LOV group (all evaluation times) | | | | | | | | | | | | |
